# Urothelial Calcium-Sensing Receptor Modulates Micturition Function via Mediating Detrusor Activity and Ameliorates Bladder Hyperactivity in Rats

**DOI:** 10.3390/ph14100960

**Published:** 2021-09-23

**Authors:** Wei-Yi Wu, Shih-Pin Lee, Bing-Juin Chiang, Wei-Yu Lin, Chiang-Ting Chien

**Affiliations:** 1 Department of Life Science, School of Life Science, College of Science, National Taiwan Normal University, Taipei 11677, Taiwan; wayne19000@gmail.com; 2 Department of Public Health, International College, Krirk University, Bangkok 10220, Thailand; cornelius.lee@gmail.com; 3 College of Medicine, Fu-Jen Catholic University, New Taipei City 24205, Taiwan; 4 Department of Urology, Cardinal Tien Hospital, New Taipei City 23148, Taiwan; 5 Department of Urology, Ministry of Health and Welfare, Taipei Hospital, New Taipei City 24213, Taiwan

**Keywords:** calcium-sensing receptor, chemosensory, detrusor activity, micturition, urothelium

## Abstract

The urothelium displays mechano- and chemosensory functions via numerous receptors and channels. The calcium-sensing receptor (CaSR) detects extracellular calcium and modulates several physiological functions. Nonetheless, information about the expression and the role of CaSR in lower urinary tract has been absent. We aimed to determine the existence of urothelial CaSR in urinary bladder and its effect on micturition function. We utilized Western blot to confirm the expression of CaSR in bladder and used immunofluorescence to verify the location of the CaSR in the bladder urothelium via colocalization with uroplakin III A. The activation of urothelial CaSR via the CaSR agonist, AC-265347 (AC), decreased urinary bladder smooth muscle (detrusor) activity, whereas its inhibition via the CaSR antagonist, NPS-2143 hydrochloride (NPS), increased detrusor activity in in vitro myography experiments. Cystometry, bladder nerve activities recording, and bladder surface microcirculation detection were conducted to evaluate the effects of the urothelial CaSR via intravesical administrations. Intravesical AC inhibited micturition reflex, bladder afferent and efferent nerve activities, and reversed cystitis-induced bladder hyperactivity. The urothelial CaSR demonstrated a chemosensory function, and modulated micturition reflex via regulating detrusor activity. This study provided further evidence of how the urothelial CaSR mediated micturition and implicated the urothelial CaSR as a potential pharmacotherapeutic target in the intervention of bladder disorders.

## 1. Introduction

Urine contains several biologically active products, which may participate in micturition function beyond the excretion of metabolic waste [1]. The alterations of urine components, pH value, and osmolality caused by multiple circumstances provoke micturition reflex in patients with lower urinary tract symptoms, such as urgency, frequency, and pelvic pain, with various scales during the course of a day [2].

The micturition cycle is composed of a voiding phase with urinary bladder smooth muscle, also known as the detrusor, contraction for bladder emptying mediated by the parasympathetic nerve, a storage phase with detrusor relaxation for bladder filling controlled by the sympathetic nerve, and bladder pressure sensation contributed by sensory neurons within the bladder wall [3]. Micturition reflex is coordinated by the spinobulbospinal system via transmitting ascending signal from the afferent pelvic nerve regarding bladder filling to periaqueductal grey and pontine micturition center, and subsequently eliciting the parasympathetic nerve-mediated contraction of detrusor [4].

Recent evidence indicates that urothelium, a highly specialized epithelium lining the lower urinary tract, possesses specialized mechanosensory and chemosensory function beyond barrier defense against urine substances [3,5,6]. Numerous evidence demonstrate that the urothelium acts as the sensory web, which receives mechanical and chemical stimuli, and afterward transduces signals to adjacent tissues for the modulation of micturition function [7]. The urothelium achieves mechanosensory function via sensing the stretch of the bladder wall and the increased intravesical pressure, and afterward releasing signaling factors, such as adenosine triphosphate, to activate the afferent nerves in suburothelium for conveying the filling information to the central nerve system [8]. Moreover, the urothelium expresses numerous receptors and ion channels, including purinergic, nicotinic, muscarinic, adrenergic receptors, and transient receptor potential ion channels, which sense chemical stimuli, including ions, adenosine, prostaglandins, and even drugs. Subsequently, the urothelium releases mediators, including adenosine, acetylcholine, neuropeptides, nitric oxide, and H_2_S to initiate the intercellular communications for the direct or indirect modulation of micturition function [9,10]. These sensory and impermeable characteristics of the urothelium achieve the benefits of drug delivery via intravesical administration that provide therapeutic effect on urinary tract disorder and avoids the systemic distribution of drugs [6,11].

The calcium-sensing receptor (CaSR) is a G-protein-coupled receptor that senses extracellular calcium and regulates Ca^2+^ homeostasis predominantly in the parathyroid glands and kidneys. The CaSR is also expressed in other tissues, such as lungs and vasculature, where it regulates non-calcitropic actions including cardiac and lung function [12]. Previous research also provides evidence that the CaSR in endothelium regulates vascular tone via nitric oxide synthesis and transient receptor potential channels [13,14]. Furthermore, the CaSR in submucosal plexus and myenteric plexus decreases enteric nerve activity and inhibits gut motility [15]. Urinary calcium levels vary broadly with numerous factors including hormones, metabolic status, dietary intake, and diseases [16]. Therefore, we hypothesized that urinary calcium might display a specific role in micturition function. However, the evidence of the expression and the role of CaSR in lower urinary tract was still absent. Consequently, we aimed to determine the existence of CaSR in urinary bladder and its pharmacophysiological significance to the micturition modulation.

In this study, we prospectively determined the expression of CaSR in the bladder urothelium of rats. We assessed how the CaSR influences bladder function with in vitro myography of bladder strips, cystometry, recording of bladder nerve activities, and bladder surface microcirculation detection. Furthermore, we evaluated the effect of the CaSR on cystitis-induced bladder hyperactivity. We characterized the role of the CaSR in bladder urothelium, elucidated the modulation of micturition function via the urothelial CaSR, and implicated urothelial CaSR as a potential pharmacological target in the treatment of bladder disorders.

## 2. Results

### 2.1. Expression of CaSR in Bladder

**Figure 1:**
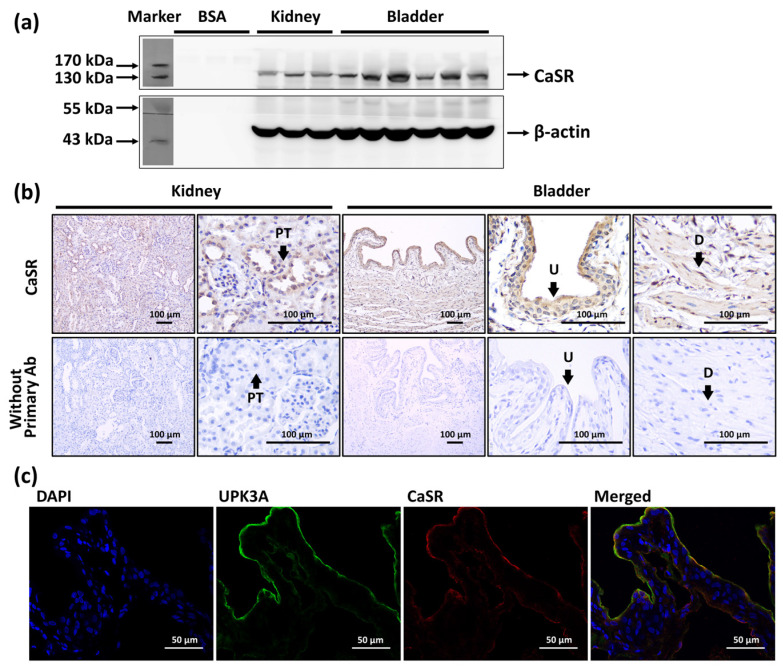
The expression and location of calcium-sensing receptor (CaSR) in bladder. (**a**) Western blot analysis of CaSR (MW: 130 kDa) in the whole kidney and whole urinary bladder. (**b**) Immunohistochemistry staining of CaSR (brown signals) in the urothelium (U) and detrusor (D) of bladder, and in the proximal tubule (PT) of kidney, and the staining without applying primary antibody (Ab). (**c**) Immunofluorescence staining of CaSR (red), uroplakin III A (UPK3A) (green), and DAPI (blue) in urothelium of urinary bladder.

The presence of the calcium-sensing receptor (CaSR) in the rat urinary bladder was demonstrated using Western blot (Figure 1a). Bovine serum albumin (BSA) was selected as a negative control, and the whole kidney was selected as a positive control because of its well-known expression of CaSR [17]. We utilized immunohistochemistry staining to confirm the location of CaSR in urinary bladder. Our result demonstrated that the CaSR was slightly expressed in the detrusor layer, whereas it was abundantly expressed in the stratified bladder urothelium. Kidney sections were utilized as a positive control, in which the CaSR was expressed in the renal tubule. Staining without applying primary antibodies was conducted to ensure the observed staining was not caused by the detection system or the specimens (Figure 1b). Furthermore, we ensured the location of the CaSR by immunolocalization of CaSR and uroplakin III A (UPK3A) (Figure 1c), which is one of the specific differentiation products of urothelial cells contributing to the urothelium barrier [18]. We evidenced that the CaSR was colocalized with UPK3A and expressed in the urothelium.

### 2.2. CaSR Influenced In Vitro Myogenic Spontaneous Activity

**Figure 2:**
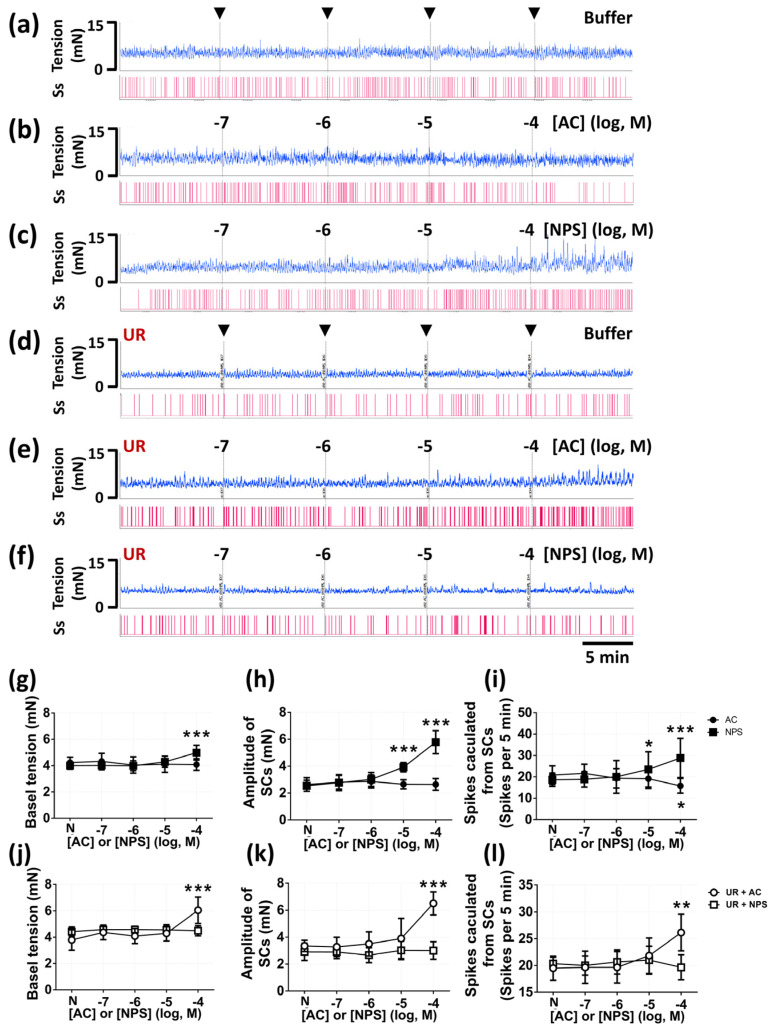
The effects of CaSR agonist (AC-265347, AC) and CaSR antagonist (NPS-2143 hydrochloride, NPS) on myogenic spontaneous activity *in vitro*. The recording of intact bladder strips myogenic spontaneous activity added with buffer (**a**), AC (from 1 × 10^−7^ to 1 × 10^−4^ M) (**b**), and NPS (from 1 × 10^−7^ to 1 × 10^−4^ M) (**c**). The recording of urothelium removal (UR) bladder strips added with buffer (**d**), AC (from 1 × 10^−7^ to 1 × 10^−4^ M) (**e**), and NPS (from 1 × 10^−7^ to 1 × 10^−4^ M) (**f**). The statistical analysis of the basal tension (**g**), amplitude of spontaneous contractions (SCs) (**h**), and spikes calculated from SCs (**i**) in intact bladder strips, and the basal tension (**j**), amplitude of spontaneous contractions (SCs) (**k**), and spikes calculated from SCs (**l**) in urothelium removal bladder strips that were affected by AC and NPS (Two-way ANOVA with Sidak’s post hoc tests, *n* = 6 for each group; ** *p* < 0.01, *** *p* < 0.001, as compared with the normal status of each strip) (N, normal status).

We evaluated the actions of the CaSR agonist (AC-265347, AC) and the CaSR antagonist (NPS-2143 hydrochloride, NPS) on in vitro bladder strips. We examined the dose-dependent effects of AC and NPS (from 10^−7^ to 10^−4^ M) on spontaneous contractions (SCs) and smooth muscle tension in intact bladder strips (Figure 2b,c) and urothelium removal (UR) bladder strips (Figure 2e,f), while the control group of intact strips and urothelium removal strips was added with Krebs–Henseleit buffer (KH buffer) in accordance with the timeline of treatments to ensure that these alterations were irrelevant to the duration of experiments (Figure 2a,d). In the groups of the intact bladder strips (*n* = six per group), our results demonstrated that NPS (100 μM) significantly increased the basal tension (4.98 ± 0.56 vs. 4.00 ± 0.27 mN, *p* < 0.0001), whereas AC showed no effect (*p* = 0.8527 in 10^−4^ M), as compared with the normal status (N) of each strip (Figure 2g). In addition, NPS (10 and 100 μM) significantly increased the amplitude of SCs (3.92 ± 0.34 vs. 2.54 ± 0.40, *p* < 0.0001; 4.78 ± 0.79 vs. 2.54 ± 0.40 mN, *p* < 0.0001), whereas AC possessed no effect on the amplitude of SCs (all *p* > 0.9999), as compared with the normal status (N) (Figure 2h). We subsequently evaluated the number of spikes per 5 min calculated from SCs. NPS (10 and 100 μM) significantly increased the number of spikes (23.5 ± 8.21 vs. 18.6 ± 3.07, *p* = 0.0297; 28.8 ± 9.19 vs. 18.6 ± 3.07, *p* < 0.0001), and AC (100 μM) significantly decreased the number of spikes (15.8 ± 3.43 vs. 20.8 ± 4.35, *p* = 0.0374), as compared with the normal status (N) (Figure 2i). Interestingly, in the groups of the urothelium removal (UR) bladder strips (*n* = six per group), we found that AC (100 μM) significantly increased the basal tension (6.05 ± 1.08 vs. 3.79 ± 0.77 mN, *p* < 0.0001) (Figure 2j), the amplitude of SCs (6.51 ± 0.86 vs. 3.35 ± 0.42 mN, *p* < 0.0001) (Figure 2k), and the number of spikes (26.16 ± 3.43 vs. 19.50 ± 2.25, *p* = 0.0022) (Figure 2l), as compared with the normal status (N). Moreover, NPS did not demonstrate obvious effect on smooth muscle tension, the amplitude of SCs, and the number of spikes in the urothelium removal (UR) bladder strips (all *p* > 0.9999), as compared with the normal status.

### 2.3. CaSR Involved in In Vitro Acetylcholine-Induced Contraction

**Figure 3:**
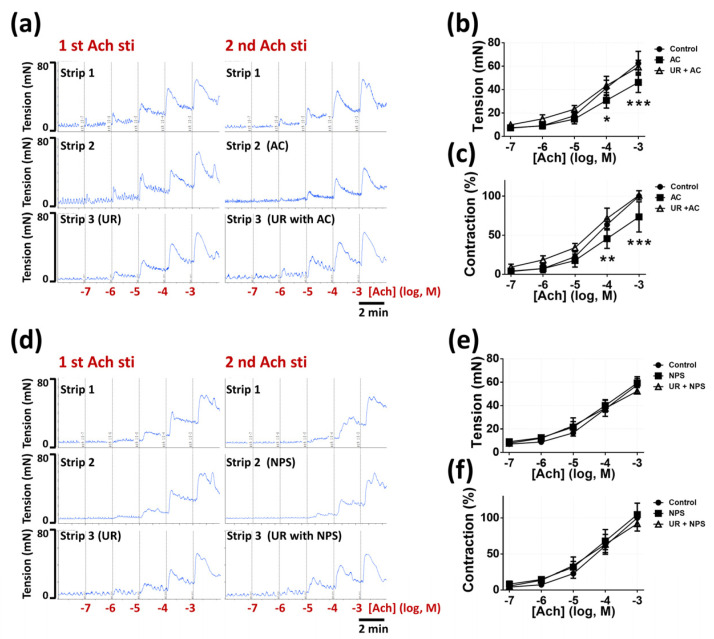
The effects of CaSR agonist (AC) and CaSR antagonist (NPS) on bladder strips contraction. The recording of contraction in response to the first and the second time of acetylcholine stimulations (sti) (from 1 × 10^−7^ to 1 × 10^−3^ M) in each group, which were added with different treatments before the second time of stimulations. (**a**) Contraction responses before and after added with buffer (control) (strip 1, intact strips) or 0.1 mM AC (strip 2, intact strips) or 0.1 mM AC (strip 3, urothelium removal strips). (**d**) Contraction responses before and after added with buffer (control) (strip 1, intact strips) or 0.1 mM NPS (strip 2, intact strips) or 0.1 mM NPS (strip 3, urothelium removal strips). The statistical analysis of contraction curves affected by AC (**b**,**c**) and NPS (**e**,**f**) in intact and urothelium removal strips (Two-way ANOVA with Sidak’s post hoc tests, *n* = 6 for each group; * *p* < 0.05, ** *p* < 0.01, *** *p* < 0.001, as compared with the control group).

We assessed the involvement of the CaSR agonist (AC) and the CaSR antagonist (NPS) in in vitro acetylcholine-induced contraction. All the bladder strips underwent the first process of acetylcholine-induced contraction to ensure the normal responses to stimulations. After washing and stabilization, each strip underwent the second acetylcholine-induced contraction combined with pretreatments of KH buffer, which were set as the control group, or AC (100 μM), or NPS (100 μM) in the intact bladder strips (*n* = six per group) (Figure 3a) and the urothelium removal (UR) bladder strips (*n* = six per group) (Figure 3d). Our results demonstrated that AC (100 μM) significantly inhibited the force of contraction in response to 100 μM and 1 mM acetylcholine (30.8 ± 6.6 vs. 41.4 ± 6.5, *p* = 0.0160; 46.1 ± 8.6 vs. 62.4 ± 10.2 mN, *p* < 0.0001) (Figure 3b), which was consistent with the relative level of contraction (45.6 ± 12.4 vs. 63.5 ± 6.8, *p* = 0.0053; 73.4 ± 19.3 vs. 100%, *p* < 0.0001) (Figure 3c) in the intact bladder strips, as compared with the control group. However, in the urothelium removal (UR) bladder strips, AC possessed no effect on acetylcholine-induced contraction (*p* = 0.9957), as compared with the control group. Furthermore, our findings indicated that NPS (100 μM) displayed no effect on acetylcholine-induced contraction (Figure 3e) and the relative level of contraction (Figure 3f) in the intact bladder strips (*p* > 0.9999, *p* = 0.8348, respectively) and in the urothelium removal (UR) bladder strips (*p* > 0.9999, *p* = 0.2866, respectively), as compared with the control group.

### 2.4. Action of CaSR on Micturition in the Anaesthetized Rat

**Figure 4:**
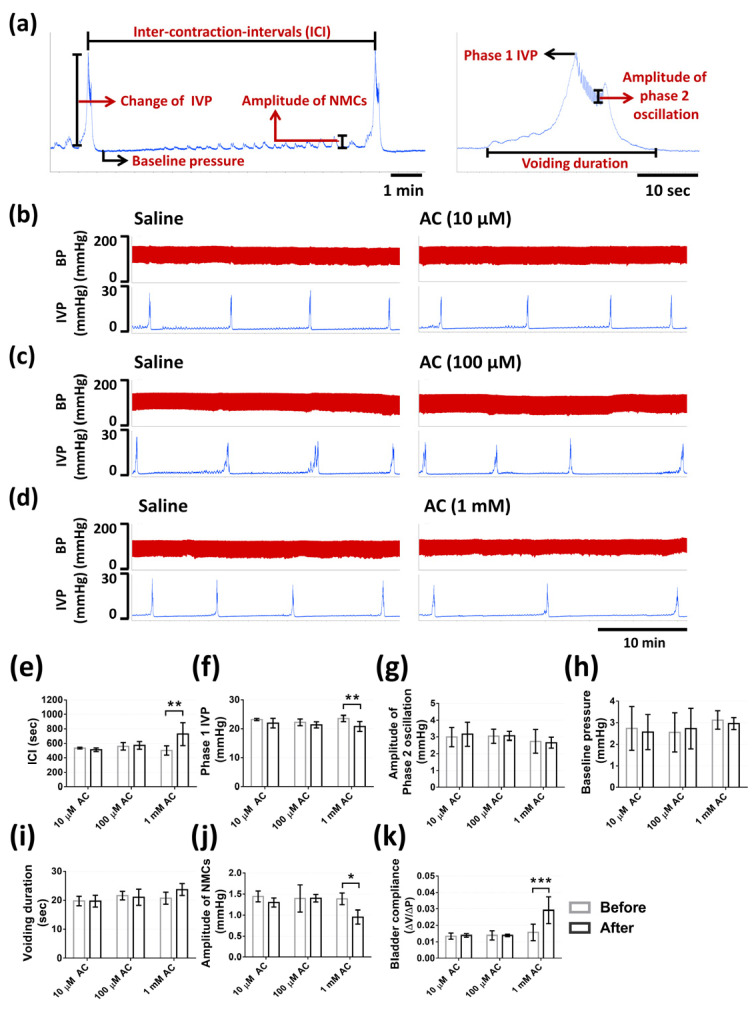
The effects of different dosages of CaSR agonist (AC) on micturition. (**a**) Illustration of micturition function parameters that were measured in this study. Effects of intravesical administrations of 10 µM AC (**b**) (*n* = 3), 100 µM AC (**c**) (*n* = 3), and 1 mM AC (**d**) (*n* = 6) on micturition with the recording of intravesical pressure (IVP) and blood pressure (BP). The statistical analysis of micturition function parameters before (saline) and after different treatments, including inter-contraction-intervals (ICI) (**e**), phase 1 IVP (**f**), amplitude of phase 2 oscillation (**g**), baseline pressure (**h**), voiding duration (**i**), amplitude of non-micturition contractions (NMCs) (**j**), and bladder compliance (**k**) (Two-way ANOVA with Sidak’s post hoc tests; * *p* < 0.05, ** *p* < 0.01, *** *p* < 0.001, as compared with saline treatment in each group).

The results from continuous recording of intravesical pressure (IVP) demonstrated the actions of CaSR on bladder urodynamics through different treatments as described in the methods. Rats were intravesically infused with saline to ensure the normal voiding responses to the bladder infusion and were set as the baseline of micturition function parameters for each group. Afterward, in the first part, intravesical administrations of 10 µM (*n* = 3), 100 µM (*n* = 3), and 1 mM CaSR agonist (AC) (*n* = 6) were applied to ascertain the dosage that can cause significant variations. Intravesical administrations of 10 and 100 µM AC did not cause obvious response (Figure 4b,c), whereas 1 mM AC demonstrated apparent inhibition of voiding responses (Figure 4d). Intravesical 10 and 100 µM AC demonstrated no effect on inter-contraction-intervals (ICI), phase one IVP, the amplitude of phase two oscillation, baseline pressure, voiding duration, amplitude of non-micturition contractions (NMCs), bladder compliance (*p* = 0.9875, *p* = 0.5978, *p* = 0.9737, *p* = 0.9852, *p* > 0.9999, *p* = 0.6617, *p* = 0.9996, sequentially, in 10 µM AC treatment; *p* = 0.9977, *p* = 0.811, *p* > 0.9999, *p* = 0.9844, *p* = 0.9827, *p* > 0.9999, *p* > 0.9999, sequentially, in 100 µM AC treatment). Intravesical infusion with 1 mM AC significantly increased ICI (728.8 ± 157.9 vs. 502.3 ± 65.2 s, *p* = 0.0017), bladder compliance (0.029 ± 0.008 vs. 0.02 ± 0.004 mL × mmHg^−1^, *p* = 0.0008), and significantly decreased phase one IVP (20.7 ± 1.73 vs. 23.5 ± 1.06 mmHg, *p* = 0.0061), the amplitude of NMCs (0.95 ± 0.16 vs. 1.38 ± 0.14, *p* = 0.01), and did not alter the amplitude of phase two oscillation (*p* = 0.9914), baseline pressure (*p* = 0.9664), voiding duration (*p* = 0.9827), as compared with the saline treatment (Figure 4e–k).

**Figure 5:**
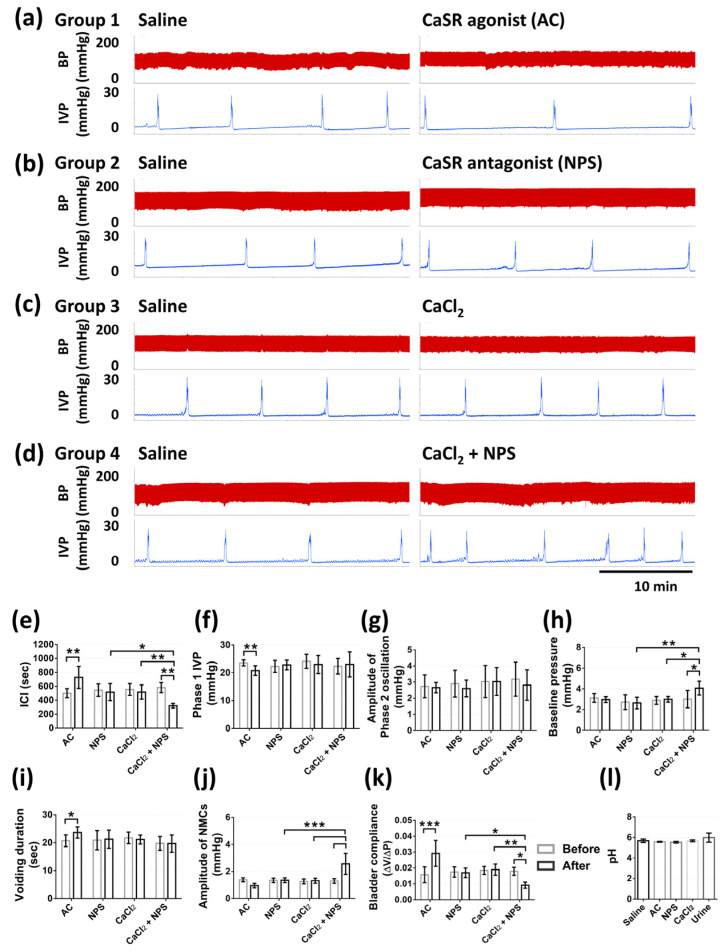
The effects of CaSR agonist (AC), CaSR antagonist (NPS), CaCl_2_, and CaCl_2_ containing NPS on micturition. Effects of intravesical administrations of 1 mM AC (**a**), 1 mM NPS (**b**), 20 mM CaCl_2_ (**c**), and 20 mM CaCl_2_ containing 1 mM NPS (CaCl_2_ + NPS) (**d**) on micturition with the recording of IVP and BP. The statistical analysis of micturition function parameters before (saline) and after different treatments, including ICI (**e**), phase 1 IVP (**f**), amplitude of phase 2 oscillation (**g**), baseline pressure (**h**), voiding duration (**i**), amplitude of NMCs (**j**), and bladder compliance (**k**). The statistical analysis of pH values of solutions for intravesical infusion and urine from rats (**l**) (Two-way ANOVA with Sidak’s post hoc tests, *n* = 6 for each group; * *p* < 0.05, ** *p* < 0.01, *** *p* < 0.001, as compared with saline treatment in each group or among different treatments).

In the second part of the experiment, intravesical administrations of 1 mM CaSR agonist (AC), 1 mM CaSR antagonist (NPS), 20 mM CaCl_2_, and 20 mM CaCl_2_ containing 1 mM CaSR antagonist (CaCl_2_ + NPS) were given, respectively, in four groups (*n* = six per group). The intravesical administration of AC inhibited voiding responses, the intravesical administration of NPS or CaCl_2_ did not induce obvious alterations, whereas the administration of CaCl_2_ containing NPS provoked voiding responses (Figure 5a–d). Intravesical AC significantly increased ICI (728.8 ± 157.9 vs. 502.3 ± 65.2 s, *p* = 0.0070), voiding duration (23.7 ± 2.02 vs. 20.7 ± 2.10 s, *p* = 0.0248), bladder compliance (0.029 ± 0.008 vs. 0.02 ± 0.004 mL × mmHg^−1^, *p* < 0.0001), and significantly decreased phase one IVP (20.7 ± 1.73 vs. 23.5 ± 1.06 mmHg, *p* = 0.0077), and showed no effect on baseline pressure (*p* = 0.9995) or the amplitude of NMCs (*p* = 0.2895), as compared with the saline treatment. Intravesical NPS or CaCl_2_ did not significantly alter these parameters, including ICI, phase one IVP, baseline pressure, voiding duration, the amplitude of NMCs, bladder compliance (*p* > 0.9999, *p* = 0.937, *p* > 0.9999, *p* > 0.9999, *p* > 0.9999, *p* > 0.9999, sequentially, in NPS treatment; all *p* > 0.9999 in CaCl_2_ treatment). Furthermore, intravesical CaCl_2_ containing NPS significantly decreased ICI (321.8 ± 32.8 vs. 582.3 ± 71.3, 517.5 ± 123.2, 518.8 ± 102.4 s, *p* = 0.0011, *p* = 0.0347, *p* = 0.0047) and bladder compliance (0.009 ± 0.0019 vs. 0.017 ± 0.0025, 0.017 ± 0.0029, 0.018 ± 0.0035 mL × mmHg^−1^, *p* = 0.0162, *p* = 0.0438, *p* = 0.0048), and significantly increased baseline pressure (4.02 ± 0.73 vs. 3.01 ± 0.83, 2.63 ± 0.55, 2.98 ± 0.27 mmHg, *p* = 0.0369, *p* = 0.0013, *p* = 0.0298) and the amplitude of NMCs (2.56 ± 0.77 vs. 1.29 ± 0.17, 1.34 ± 0.16, 1.26 ± 0.18 mmHg, all *p* < 0.0001), as compared with the saline treatment, NPS treatment, and CaCl_2_ treatment, respectively, and showed no effect on phase one IVP (*p* = 0.875) or voiding duration (*p* < 0.9999), as compared with the saline treatment. All the treatments demonstrated no effect on the amplitude of phase two oscillation (*p* = 0.9957, *p* = 0.6625, *p* > 0.9999, *p* = 0.4963 in AC, NPS, CaCl_2_, and CaCl_2_ + NPS treatments, respectively) (Figure 5e–k).

### 2.5. Effects of CaSR on Bladder Nerve Activities

**Figure 6:**
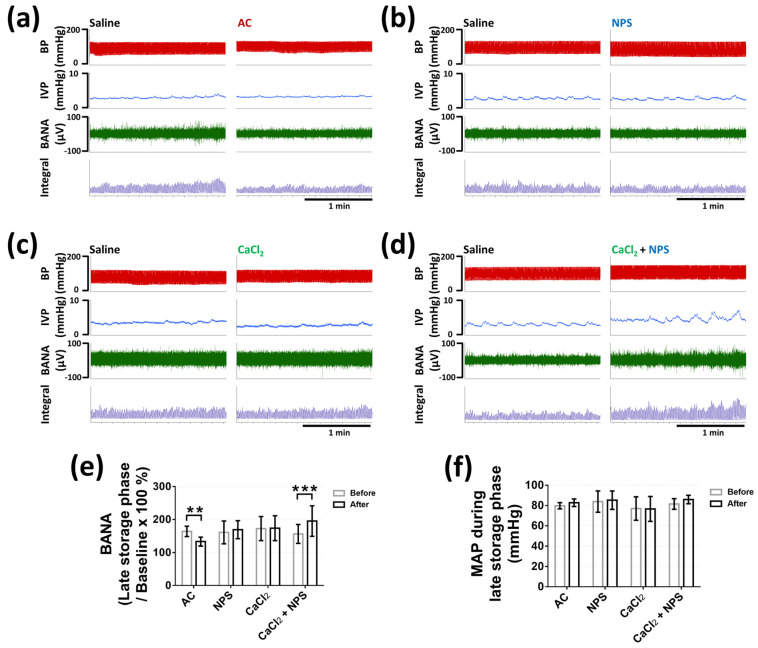
The effects of CaSR agonist (AC), CaSR antagonist (NPS), CaCl_2_, and CaCl_2_ containing NPS on bladder afferent nerve activity (BANA). The effects of intravesical administrations of 1 mM AC (**a**), 1 mM NPS (**b**), 20 mM CaCl_2_ (**c**), and 20 mM CaCl_2_ containing 1 mM NPS (CaCl_2_ and NPS) (**d**) on BANA during late storage phase. The statistical analysis of the change of BANA during late storage phase (**e**), and the mean arterial pressure (MAP) during late storage phase (**f**) before (saline) and after different treatments in each group (Two-tailed paired Student’s *t*-test, *n* = 6 for each group; ** *p* < 0.01, *** *p* < 0.001, as compared with saline treatment in each group).

We assessed bladder nerve activities (BNA) during the voiding phase, which represented bladder efferent nerve activities (BENA), and BNA during the late storage phase, which was considered as bladder afferent nerve activities (BANA), as previously described [19,20]. Four groups of rats (*n* = six per group) were intravesically infused with saline to ensure the normal uroneuro-physiological response, and followed by different treatments, as described above. Intravesical AC significantly decreased BANA during the late storage phase (133 ± 14.13 vs. 163.9 ± 15.67%, *p* = 0.0034) (Figure 6a,e), and intravesical CaCl_2_ containing NPS significantly increased BANA during the late storage phase (195.3 ± 46.44 vs. 156.2 ± 28.75%, *p* = 0.0003), as compared with the saline treatment (Figure 6d,e). In addition, intravesical NPS or CaCl_2_ did not display obvious influence on BANA during the late storage phase (*p* = 0.7692, *p* = 0.998, respectively) (Figure 6b,c,e).

**Figure 7:**
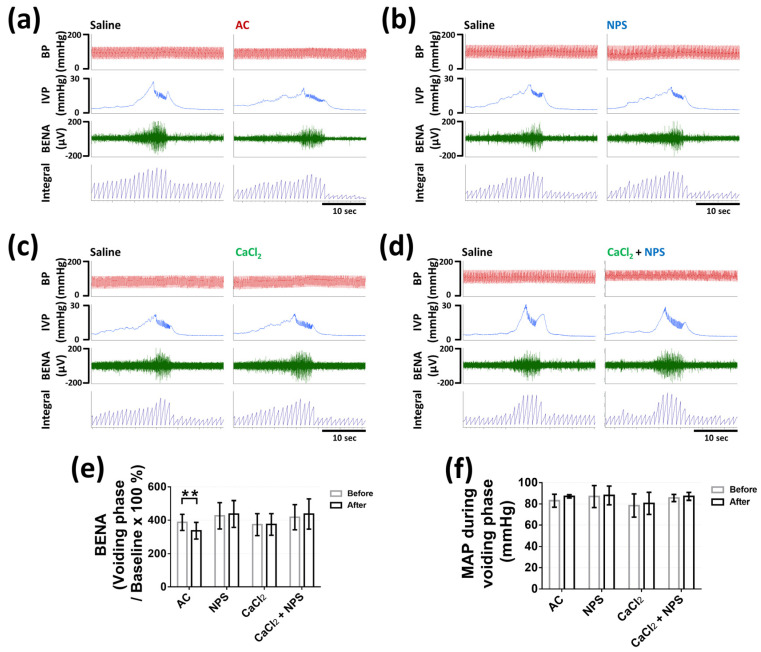
The effects of CaSR agonist (AC), CaSR antagonist (NPS), CaCl_2_, and CaCl_2_ containing NPS on bladder efferent nerve activity (BENA). The effects of intravesical administrations of 1 mM AC (**a**), 1 mM NPS (**b**), 20 mM CaCl_2_ (**c**), and 20 mM CaCl_2_ containing 1 mM NPS (CaCl_2_ + NPS) (**d**) on BENA during voiding phase. The statistical analysis of the change of BENA (**e**) and the mean arterial pressure (MAP) (**f**) before (saline) and after different treatments in each group during voiding phase (Two-tailed paired Student’s *t*-test, *n* = 6 for each group; ** *p* < 0.01, as compared with saline treatment in each group).

Moreover, intravesical 1 mM AC significantly decreased BENA during voiding (336.4 ± 50.36 vs. 387.1 ± 48.86%, *p* = 0.0002), as compared with the saline treatment (Figure 7a,e). Intravesical 1 mM NPS or 20 mM CaCl_2_ or 20 mM CaCl_2_ containing 1 mM NPS did not demonstrate noticeable effect on BENA during voiding (*p* = 0.7448, *p* = 0.999, *p* = 0.2525, respectively) (Figure 7b,e). All the intravesical treatments demonstrated no effect on the mean arterial pressure during the late storage phase (*p* = 0.3008, *p* = 0.8804, *p* = 0.9985, *p* = 0.0675 in AC, NPS, CaCl_2_, and CaCl_2_ + NPS treatments, respectively) (Figure 6f) and the voiding phase (*p* = 0.2974, *p* = 0.9847, *p* = 0.8309, *p* = 0.9364 in AC, NPS, CaCl_2_, and CaCl_2_ + NPS treatments, respectively) (Figure 7f).

### 2.6. Evaluation of Bladder Surface Microcirculation with Different Intravesical Administrations

**Figure 8:**
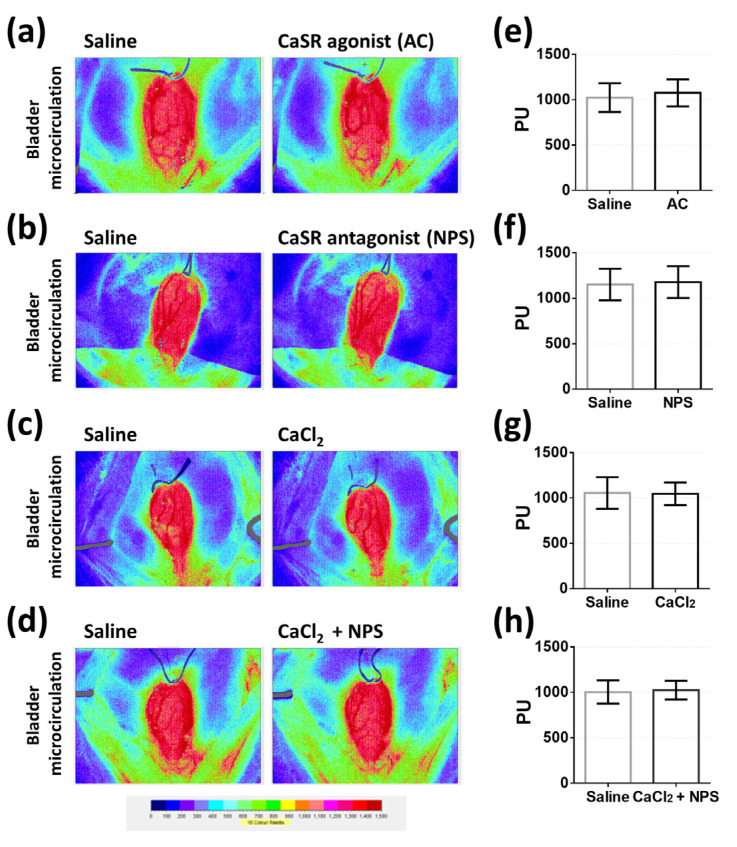
The evaluation of bladder microcirculation before and after intravesical infusion of CaSR agonist (AC), CaSR antagonist (NPS), CaCl_2_, and CaCl_2_ containing NPS. Images of bladder microcirculation before (saline) and after intravesical infusion of 1 mM AC (**a**), 1 mM NPS (**b**), 20 mM CaCl_2_ (**c**), and 20 mM CaCl_2_ containing 1 mM NPS (CaCl_2_ + NPS) (**d**). The statistical analysis of bladder perfusion unit (PU) before (saline) and after intravesical infusion of AC (**e**), NPS (**f**), CaCl_2_ (**g**), and CaCl_2_ containing NPS (CaCl_2_ + NPS) (**h**) (Two-tailed paired Student’s *t*-test, *n* = 5 for each group).

We evaluated the surface microcirculation of bladder in response to intravesical infusion with saline followed by intravesical infusion with 1 mM AC or 1 mM NPS or 20 mM CaCl_2_, or 20 mM CaCl_2_ containing 1 mM NPS in four groups (*n* = five per group), as described above. Bladder surface microcirculation before and after treatments were demonstrated in 16-color-coded images (Figure 8a–d) and perfusion units (PU). Our results demonstrated that all the treatments did not cause obvious variation in bladder surface microcirculation (*p* = 0.0827, *p* = 0.1299, *p* = 0.7478, *p* = 0.2695 in AC, NPS, CaCl_2_, and CaCl_2_ + NPS treatments, respectively) (Figure 8e–h).

### 2.7. Effect of CaSR Agonist on Bladder Hyperactivity

**Figure 9:**
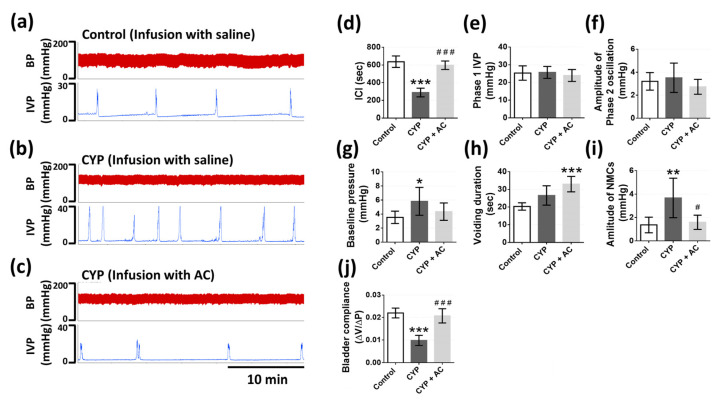
The effects of CaSR agonist (AC) on cyclophosphamide (CYP)-induced bladder hyperactivity. The recording of IVP in control group (**a**), CYP group with intravesical infusion of saline (**b**), and CYP group with intravesical infusion of AC (CYP + AC) (**c**). The statistical analysis of bladder function parameters in control group, CYP group with intravesical infusion of saline and AC, including ICI (**d**), phase 1 IVP (**e**), amplitude of phase 2 oscillation (**f**), baseline pressure (**g**), voiding duration (**h**), amplitude of NMCs (**i**), and bladder compliance (**j**) (One-way ANOVA with Tukey’s post hoc tests, *n* = 6 for each group; * *p* < 0.05, ** *p* < 0.01, *** *p* < 0.001, as compared with control group; ^#^ *p* < 0.05, ^###^ *p* < 0.001, as compared with CYP group).

We performed the continuous cystometry recording 48 h after a cyclophosphamide (CYP) injection to assess the effect of the CaSR agonist (AC) on bladder hyperactivity through the analysis of bladder function parameters, as described above. The rats were subjected to intravesical infusion with saline to confirm bladder hyperactivity was successfully induced and followed by intravesical infusion with AC (1 mM). Our results illustrated that CYP caused irregular and frequent voiding reflex, whereas intravesical AC alleviated parts of these phenomena (Figure 9a–c). From the results (Figure 9d–l), we observed that ICI (288.2 ± 49.4 vs. 637.2 ± 64.0 s, *p* < 0.0001) and bladder compliance (0.0097 ± 0.0022 vs. 0.022 ± 0.0021 mL × mmHg^−1^, *p* < 0.0001) were significantly decreased in the CYP group, as compared with the control group. Baseline pressure (5.82 ± 1.99 vs. 3.53 ± 0.88 mmHg, *p* = 0.038) and the amplitude of NMCs (3.67 ± 1.69 vs. 1.36 ± 0.66 mmHg, *p* = 0.0066) were significantly increased in the CYP group, as compared with the control group. Nevertheless, intravesical AC significantly improved some parameters of bladder hyperactivity, including ICI (597.7 ± 47.8 vs. 288.2 ± 49.4 s, *p* < 0.0001), the amplitude of NMCs (1.58 ± 0.60 vs. 3.67 ± 1.69 mmHg, *p* = 0.0135), and bladder compliance (0.0207 ± 0.0031 vs. 0.0097 ± 0.0022 mL × mmHg^−1^, *p* < 0.0001), as compared with the CYP group before intravesical infusion of AC.

## 3. Discussion

In the current study, we evidenced the expression of the CaSR in urinary bladder of rats, which was colocalized with UPK3A and was mainly expressed on the stratified urothelial cells in the bladder. In the in vitro myography experiments, we observed that the activation of urothelial CaSR via CaSR agonist (AC) inhibited the myogenic spontaneous activity and the acetylcholine-induced contraction of bladder strips. In the in vivo experiments on anaesthetized rats, we observed that the intravesical infusion with AC decreased the bladder afferent nerve activities during the late storage phase and the bladder efferent nerve activities during the voiding phase. We evidenced that these alterations of myogenic and neuronal activities via intravesical AC resulted in micturition modulation, which significantly increased inter-contraction-intervals, voiding duration, bladder compliance, and decreased phase one intravesical pressure and amplitude of non-micturition contractions. Furthermore, the activation of urothelial CaSR reversed CYP-induced bladder hyperactivity, which implicated the potential of urothelial CaSR as a therapeutic target.

In the present study, we evaluated the responses of AC-265347 with numerous ranges in in vitro myography and cystometry. We confirmed the concentration of AC-265347 that can cause noticeable variations in in vitro myography (0.1 mM) and cystometry (1 mM), whereas these dosages were higher than the dosage used in previous research, such as intraintestinal perfusion with 2 µM AC-265347 [21]. A similar situation, where urothelium is less sensitive to intravesical drug administration, also happened in other experiments. The dosage of the intravesical infusion with vibegron to activate β3-adrenoceptor in urothelium was also up to 1 mM, which was also higher than the dosage used in cellular models [22]. These circumstances may have resulted from the tight junction of the urothelium and the glycosaminoglycan layer locating at bladder luminal surface, which resulted in the bladder permeability barrier restricting the diffusion of intravesical drug administration [23].

The CaSR detects the circulating calcium concentration and influences systemic calcium homeostasis, whereas it also regulates other functions, such as vascular smooth muscle tension [12]. However, research regarding the role of the CaSR in lower urinary tract has remained absent. Consequently, we speculated the existence of the CaSR in urinary bladder and evaluated the effects of the CaSR on micturition. We explored the expression of CaSR in the bladder urothelium through Western blotting and immunohistochemistry with antibodies, of which the specificity for rat tissues were confirmed in previous research [24,25]. Our data firstly revealed the characteristic of the CaSR in bladder urothelium. Based on the results, intravesical activation of the CaSR via CaSR agonist (AC) increased ICI, voiding duration, and bladder compliance, which revealed its inhibitory effect on the micturition reflex. The intravesical infusion of the CaSR antagonist (NPS-2143 hydrochloride, NPS) did not cause apparent alterations, which proved that the CaSR acts as a chemosensory receptor in bladder and may not be involved in the mechanosensory response during micturition cycle. Based on previous research, AC-265347 is about 20-fold more potent than Ca^2+^ to the CaSR [24]. Consequently, we selected 20 mM CaCl_2_ in cystometry to determine whether it mimicked the responses of the intravesical infusion with 1 mM AC. However, the intravesical infusion of CaCl_2_ did not affect micturition reflex, which was opposite to our presumption. Consequently, we further examined the responses of the intravesical infusion of CaCl_2_ containing NPS. Intriguingly, this treatment decreased ICI and bladder compliance, and increased baseline pressure and the amplitude of NMCs, which had similarity with the patterns of urinary disorders, such as bladder hyperactivity. Moreover, we verified there was no significant difference of pH value in these solutions for intravesical infusion and the urine collected from rats, which proved these alterations were irrelevant to the variations of pH values.

It is believed that the stimulated urothelium transmits signal molecules to interstitial cells of Cajal existing in lamina propria, which is proven to form a functional syncytium with the detrusor to integrate signals, communicate with adjacent cells, and modulate micturition function [26]. Interstitial cells demonstrate spontaneous electrical activity that does not contribute to voiding response but can modify neurotransmission and the spontaneous activity of detrusor [9]. In current study, we assessed the role of the CaSR in in vitro detrusor activity to exclude the regulation from the central nervous system (CNS). Due to the inappropriateness of adjustments to the osmolality or ion concentration of the Krebs–Henseleit buffer in in vitro myography, we only applied AC and NPS in this experiment. From our data with intact bladder strips, we verified that the activation of the CaSR in bladder strips decreased the frequency of spontaneous contractile activity. Otherwise, the inhibition of the CaSR increased detrusor tension and the amplitude and the frequency of spontaneous contractions. Furthermore, activation of the CaSR inhibited acetylcholine-induced contraction, which demonstrated a similar tendency to cystometry results.

Intriguingly, the urothelium-removed bladder strips treated with AC showed the increased detrusor tension and amplitude and frequency of spontaneous contractions. These results further proved that the CaSR in detrusor smooth muscle cell possesses a stimulative effect on detrusor spontaneous activity, which is opposite to the urothelial CaSR. A similar mechanism is demonstrated that the functions of the CaSR in vascular smooth muscle cell are contrary to endothelial cell [13]. Using the method of the intact bladder strip and the urothelium removal bladder strip, we proved that the activation of urothelial CaSR predominantly mediated the inhibitory effect on detrusor activity. Moreover, under the condition of CaSR inhibition, the intact bladder strips demonstrated elevated detrusor activity, and the urothelium removal bladder strips showed no variation. These results further confirmed that the Ca^2+^ in KH buffer stimulated detrusor activity via the urothelium, which were analogous to the enhanced NMCs in cystometry data with intravesical infusion of CaCl_2_ containing NPS. Furthermore, our results implied that the urothelial CaSR suppressed the generation of NMCs via modulating adjacent cells including detrusor cell, whereas further verifications were required to elaborate these intercellular interactions.

Based on previous research, the urothelium transduces sensory signals, mediates adjacent tissues, and ultimately affects the CNS to modulate micturition [3]. For instance, activations of urothelial mechanoreceptors or chemoreceptors during the storage phase induce the release of mediators from urothelial cells, which contribute to the enhanced NMCs that provoke afferent nerve activity for bladder sensation, and afterward convey afferent outflow to the CNS for the regulation of micturition [27,28,29]. In present study, we assessed the role of urothelial CaSR in BNA during the late storage phase and voiding phase. The activation of urothelial CaSR decreased BANA during the late storage phase, in accompany with the decreased amplitude of NMCs, and subsequently decreased BENA during voiding. Intravesical infusion of CaCl_2_ or NPS conferred no effect on BNA, which was consonant with the cystometry data. Moreover, intravesical infusion of CaCl_2_ containing NPS increased BANA during the late storage phase in accompany with the increased amplitude of NMCs.

A similar tendency was demonstrated in the in vitro bladder strip results, in which the urothelial CaSR activation predominantly suppressed detrusor activity, whereas the urothelial CaSR inhibition resulted in the stimulated detrusor activity. Furthermore, we observed no significant variation of mean arterial pressure during the intravesical infusion with CaSR agonist or antagonist. The activation of urothelial CaSR did not induce global autonomic neuronal activities enhancement, which indicated the effects of these intravesical administrations on the urothelium were confined locally in bladder tissue and micturition reflex circuit. Therefore, we concluded that the activation of urothelial CaSR contributed to the inhibitory effect on micturition via inhibiting the conformation of NMCs, which subsequently decreased the afferent outflow. By contrast, with the inhibited urothelial CaSR, urinary Ca^2+^ provoked micturition via augmenting NMCs, which afterward increased the afferent activity, whereas it required further verifications of underlying mechanism.

According to previous research, the activation of endothelial CaSR induces vasodilation in mice aorta [13], which is similar to our finding in the activation of urothelial CaSR. The activation of endothelial CaSR induces the endothelium nitric oxide synthase pathway and the activation of intermediate conductance Ca^2+^-activated K^+^ (IK_Ca_) channels, which requires Ca^2+^ influx pathway possibly mediated by the transient receptor potential (TRP) superfamily of Ca^2+^-permeable cation channels [14]. Based on previous evidence, TRP channels (TRPs), which also exist mainly within the urothelium, include selective and non-selective cation channels that can be activated by several stimuli, including heat, stress, osmolality, and lipid ligands [30].

Our results demonstrated that the intravesical infusion of CaCl_2_ did not demonstrate the same effects of the intravesical infusion of AC on micturition. Moreover, when the urothelial CaSR was inhibited by NPS, intravesical infusion of CaCl_2_ caused remarkably differences of micturition parameters, as compared with the intravesical infusion of AC. Therefore, we speculated that the urinary Ca^2+^ not only activated the CaSR, but also may be involved in unspecific activations of TRPs or the other targets, which facilitate voiding reflex, whereas the urothelial CaSR may be involved in the inhibitory regulation against those unspecific activations. We hypothesized that the CaSR may participate in the rebalancing mechanism against the excitation caused by the abrupt increase of calcium concentration in urine. When the CaSR is inhibited by NPS, urinary Ca^2+^ may non-specifically stimulate voiding reflex through these mechanisms. Nonetheless, further research is required to clarify the detailed targets that are activated by urinary Ca^2+^ to provoke voiding reflex, and the interactions of CaSR with other receptors or channels in the urothelium.

The vascular endothelial CaSR mediates vascular tension and circulation through the vasodilatation effect on the arterioles, such as the mesenteric artery [13]. Due to the impermeable characteristic of urothelium, the intravesical administration of drugs may not directly influence the vascular tension of bladder vasculature. Nonetheless, we did not assure whether the signaling molecules released from urothelium would indirectly affect the vascular smooth muscle cells of bladder vasculature through adjacent cells, as previously proposed [31]. According to results, all the intravesical treatments did not affect the microcirculation of bladder tissue. We proved that the modulation of micturition function via the urothelial CaSR was not correlated with hemodynamic disturbance in the bladder.

The functions of bladder urothelium have not only been considered as the physical barrier, but also the sensation that responds to multiple stimuli, and serves as the transducer for transmitting sensory signaling to adherent tissue to modulate micturition function [1,3,32]. For instance, a previous study proves the activation of beta 3-adrenoceptor, which is also expressed in the urothelium, improves bladder storage via inhibiting bladder afferent signaling and detrusor activity [33]. Intravesical administrations of purinergic agonists activate the P_2_X receptor in the urothelium, and afterward, increases bladder afferent nerve activities [34]. This evidence ascertains the sensory function of urothelium and indicates urothelial receptors may be involved in the pathophysiological mechanisms of bladder disorders, such as interstitial cystitis and bladder hypersensitivity, whereas the urothelium can also be the drug target for the interventions of bladder diseases.

Therefore, we examined the effects of CaSR agonist on interstitial cystitis-induced bladder hyperactivity caused by cyclophosphamide (CYP). CYP is a well-established experimental method to induce cystitis with features of bladder pain, bladder over-activity, and bladder inflammation [35]. Our results demonstrated that CYP decreased ICI and bladder compliance, and increased baseline pressure and the amplitude of NMCs. These alterations were reversed by the intravesical administration of CaSR agonist, which indicated that the CaSR may be a pharmacotherapeutic target for the treatment of bladder hyperactivity in patients.

Our current results supported the chemosensory role of urothelial CaSR in the bladder urothelium, and we further suggested the potential clinical applications of the urothelial CaSR in micturition modulation. Nonetheless, there are some unsolved issues, including the effectiveness of long-term administration of CaSR agonist, and the role of CaSR in the pathophysiology of bladder dysfunctions induced by multiple factors. Comprehensive studies are needed to discover the effective dose of AC-265347 in the activation of urothelial CaSR with cellular models, the signaling molecules released from the urothelium via CaSR activation, the downstream targets in the signaling pathway, and subsequently, the intracellular mechanisms of signaling cascades.

In summary, our research demonstrated the existence of CaSR in the bladder urothelium of rats. We provided concrete evidence of how the urothelial CaSR mediated micturition function. Our in vivo and in vitro results elucidated that the urothelial CaSR acted as a chemosensory receptor in bladder, and its activation modulated micturition function via regulating detrusor activity, rather than hemodynamic disturbance. We confirmed that the activation of urothelial CaSR via intravesical administration with CaSR agonist (AC-265347) displayed an inhibitory effect on micturition cycle, including decreased NMCs, attenuated BANA during the storage phase and BENA during the voiding phase, and inhibited micturition reflex. Intravesical administration with CaSR agonist ameliorated cystitis-induced bladder hyperactivity, which further suggested the promising potential of the urothelial CaSR as a pharmacotherapeutic target for bladder disorders.

## 4. Materials and Methods

### 4.1. Ethical Approval

All animal experiments were performed in accordance with the ARRIVE guidelines (Animal Research: Reporting of In Vivo Experiments) and were approved by the Institutional Animal Care and Use Committee (IACUC) of National Taiwan Normal University (Number of ethical approval, 108005).

### 4.2. Animals

Female 8-week-old Sprague Dawley rats were purchased from BioLASCO Taiwan Co., Ltd., (Yi Lan, Taipei, Taiwan). Animals were housed under standard laboratory conditions with a controlled room temperature and a 12-h dark–light cycle and free access to food and tap water in the animal center of National Taiwan Normal University. After a one-week period of accommodation, rats were randomly assigned to different groups. All animal experiments were performed under anesthesia, and efforts were made to minimize suffering. After experiments, all animals were sacrificed with the intravenous injection of 3 M KCl.

### 4.3. Western Blot

The expression of CaSR in the whole bladder and kidney tissue was analyzed using Western blot in accordance with the previously described method [36]. During protein preparation, the sample was homogenized with a lysis buffer containing 100 mM iodoacetamide (Sigma-Aldrich, St. Louis, MO, USA), and the supernatant was mixed with SDS-sample buffer containing 100 mM dithiothreitol (Sigma-Aldrich, St. Louis, MO, USA). Sodium dodecyl sulfate-polyacrylamide (SDS) gel electrophoresis was performed on 10% separation gels. Protein was transferred to Immobilon polyvinylidene difluoride membranes (Millipore, Billerica, MA, USA). Whole kidney tissue was selected as a positive control to confirm the specificity of the primary antibody. Monoclonal CaSR antibody (1:100; NB100-1830; Novus Biologicals, Littleton, CO, USA) was used as the primary antibody. Horseradish peroxidase (HRP) conjugated rabbit anti-mouse IgG (1:10,000; Sigma-Aldrich, St. Louis, MO, USA) was used as the secondary antibody. The density of the band was detected using densitometry with image analysis system (Alpha Innotech, San Leandro, CA, USA).

### 4.4. Immunohistochemistry and Immunofluorescence

In immunohistochemistry staining, bladder and kidney samples were fixed in 10% buffered formalin and embedded in paraffin for histological analysis of CaSR. Sections were deparaffinized, rehydrated, performed with heat-induced antigen retrieval at 95 °C by retrieval buffer (10 mM sodium citrate, 0.05% Tween-20, pH 6.0), and incubated overnight at 4 °C with CaSR antibody (1:200; MA1-934; Thermo Fisher, Rockford, IL, USA). Sections were then applied with HRP-conjugated secondary antibody (1:800; Sigma-Aldrich, St. Louis, MO, USA), followed by chromogenic detection with diaminobenzidine (ImmPACT DAB peroxidase substrate kit, Vector Laboratory INC, Burlingame, CA, USA). In immunofluorescence staining, tissue samples were embedded with optimal cutting temperature compound. Sections were incubated with primary antibodies, including CaSR (1:100; Thermo Fisher, Rockford, IL, USA) and uroplakin III A (UPK3A) (1:50; bs-13703R; Bioss, Woburn, MA, USA), and afterward followed by incubation with fluorescent secondary antibodies, including goat anti-mouse Alexa-Fluor IgG or goat anti-rabbit FITC IgG (1:800; Abcam, Cambridge, UK). Cell nuclei were stained with 4′,6-Diamidino-2-phenylindole dihydrochloride (DAPI; Sigma-Aldrich, St. Louis, MO, USA).

### 4.5. Measurement of In Vitro Bladder Strip Contractility

We utilized the in vitro smooth muscle contractility method to evaluate the effects of urothelial CaSR activation and inhibition on the myogenic spontaneous activity and the contraction in response to acetylcholine (Sigma-Aldrich, St. Louis, MO, USA) with intact bladder strips (*n* = 6) and urothelium removal strips (*n* = 6). Specific calcimimetic, CaSR agonist (AC-265347, AC; Sigma-Aldrich, St. Louis, MO, USA), and calcilytic, CaSR antagonist (NPS-2143 hydrochloride, NPS; Sigma-Aldrich, St. Louis, MO, USA) were applied on the basis of previous evidence [24,37,38]. After rats were anaesthetized with inhalation of isoflurane, urinary bladder was immediately removed, and placed in modified Krebs–Henseleit buffer (KH buffer) (Sigma-Aldrich, St. Louis, MO, USA) of the following formula: NaCl, 114 mM; KCl, 4.7 mM; CaCl_2_, 2.5 mM; MgSO_4_, 1.2 mM; KH_2_PO_4_, 1.2 mM; NaHCO_3_, 25 mM; and glucose, 11.7 mM (maintained at 37 °C, oxygenated with 95% O_2_ and 5% CO_2_, pH = 7.4), in accordance with the well-established method [39]. Strips were cut along the circular muscle axis and mounted in muscle bath of myograph (Multi Wire Myograph System DMT 620M; Danish Myo Technology, Aarhus, Denmark) filled with KH buffer in chambers. The preparation of urothelium removal was executed with ophthalmic forceps and ophthalmic scissors under a light microscope. Strips were left to equilibrate for at least 60 min with a basal resting tension of 5 mN before experiments of spontaneous contractions and acetylcholine-induced contraction. In the experiments of spontaneous contractions, CaSR agonist (AC-265347) or CaSR antagonist (NPS-2143 hydrochloride), which were dissolved in KH buffer, were added into chambers with concentrations from 1 × 10^−7^ to 1 × 10^−4^ M. The spontaneous contractions after each concentration of CaSR agonist or CaSR antagonist treatments were observed at least for 10 min. Parameters including the tension, the number, and the amplitude of spontaneous contractions were measured. In the experiments of acetylcholine-induced contraction, CaSR agonist (1 × 10^−4^ M) or CaSR antagonist (1 × 10^−4^ M) were added into chambers 30 min before acetylcholine treatment. Acetylcholine with concentrations from 1 × 10^−7^ to 1 × 10^−3^ M were added sequentially with intervals of 2 min. The maximum contractions after each acetylcholine treatment were measured for each strip.

### 4.6. Measurement of Cystometric Parameters

We utilized the transcystometric model with female rats to measure alterations of micturition in response to different treatments, which was in compliance with the well-established method [19]. At first, rats were randomly divided into three groups with intravesical infusion of saline followed by intravesical infusion of different dosages of CaSR agonist (AC-265347, AC) (10 µM, *n* = 3; 100 µM, *n* = 3; 1 mM, *n* = 6) to evaluate which dosage can display noticeable variations. Second, rats were randomly divided into four groups, including intravesical infusion with saline followed by intravesical infusion with 1 mM CaSR agonist (AC-265347, AC) (Group 1, *n* = 6), intravesical infusion with saline followed by intravesical infusion with 1 mM CaSR antagonist (NPS-2143 hydrochloride, NPS) (Group 2, *n* = 6), intravesical infusion with saline followed by intravesical infusion with 20 mM CaCl_2_ (Sigma-Aldrich, St. Louis, MO, USA) (Group 3, *n* = 6), and intravesical infusion with saline followed by intravesical infusion with 20 mM CaCl_2_ containing 1 mM CaSR antagonist (CaCl_2_ + NPS) (Group 4, *n* = 6). All of the drugs used above were dissolved in saline. We further measured the pH values of solutions used in intravesical administrations, including saline (pH = 5.68 ± 0.16), 1 mM AC (pH = 5.58 ± 0.04), 1 mM NPS (pH = 5.53 ± 0.08), and 20 mM CaCl_2_ (pH = 5.67 ± 0.08). We also measured the pH values of urine from rats (pH = 5.99 ± 0.44) (*n* = 6). There was no significant difference among these solutions and urine samples (as shown in Figure 5l). Rats were anesthetized with subcutaneous injection of urethane (1.2 g × kg^−1^ body weight; Sigma-Aldrich, St. Louis, MO, USA), which is a preferable anesthetic agent for acute cystometric studies in rats because of its relatively modest suppression on bladder responses during cystometry [40]. Bladder was exposed through a midline incision of the abdomen. PE-50 catheter was inserted through bladder dome, and afterward catheter was connected to a pressure transducer (DPT-100; Utah Medical Products Inc., Midvale, UT, USA) and infusion pump with an infusion rate of 0.02 mL × min^−1^. Intravesical pressure (IVP) was continuously recorded using a PowerLab system (ADInstruments, Castle Hill, NSW, Australia). Parameters of bladder cystometry were measured as previously described [19], including inter-contraction-intervals (ICI), phase 1 IVP, amplitude of phase 2 oscillation, baseline pressure, voiding duration, amplitude of non-micturition contractions (NMCs), and bladder compliance (micturition volume/change of IVP) (as shown in Figure 4a). Blood pressure (BP) was measured for monitoring of vital sign via carotid artery catheter connected to the pressure transducer and PowerLab system.

### 4.7. Bladder Nerve Activities Recording

Bladder nerve activities (BNA) were measured before and after different treatments in four groups (6 rats per group) that were described above. At first, bladder was catheterized as described above in the section of transcystometric model. Multi-fiber BNA was recorded as previously described [19]. Briefly, postganglionic bladder nerves originating from the major pelvic ganglion that attached to the urinary bladder surface were isolated and the nerve activity was recorded by the placement of intact nerve fiber on a bipolar stainless steel electrode. Nerve signals were amplified 20,000-fold, filtered (high-frequency cutoff at 3000 Hz and low-frequency cutoff at 30 Hz) with AC preamplifier (Grass model P511, Valley View, OH, USA), and continuously recorded with the PowerLab system. The original nerve activity was converted to pulse spikes and was afterward integrated every 1 s by the PowerLab system. BNA at the beginning of storage phase were set as the baseline of BNA. Bladder afferent nerve activities (BANA) during late storage phase and bladder efferent nerve activities (BENA) during voiding were calculated as percentage change from the baseline value of BNA of each individual.

### 4.8. Determination of Bladder Surface Microcirculation

Continuous microcirculation intensity of whole bladder surface was recorded with a full-field laser perfusion imager (Moor FLPI, Moor Instruments, Devon, UK) [41]. Moor FLPI permits non-contact recording of blood flow in rat bladder microvasculature and uses laser speckle contrast imaging to quantify the level of movements of blood cells within the region of interest. Moor FLPI converts contrast image to 16-color-coded image and perfusion units (PU) that are correlated with the surface microcirculation intensity in the tissue using Moor FLPI 3.0 software (Moor Instruments, Devon, UK). The red region in 16-color-coded image represents high intensity of microcirculation. On the contrary, the blue region represents low intensity of microcirculation. After the bladder was exposed with previously described procedure, bladder was inserted with a PE-50 catheter through bladder dome for the intravesical infusion of saline and the following different treatments in four groups (5 rats per group) as described above. In order to avoid the interference of microcirculation caused by the overexpansion of bladder volume, urethra cannulation was conducted with another PE-50 catheter to draw liquid from bladder, avoid leaking from urethra, and consequently maintain bladder volume at 0.2 mL during recording. Microcirculation intensity was recorded to evaluate whether our treatments would produce hemodynamic disturbances in the bladder.

### 4.9. Cyclophosphamide-Induced Bladder Hyperactivity

Bladder hyperactivity was induced by a single intraperitoneal injection of cyclophosphamide (CYP; Sigma-Aldrich, St. Louis, MO, USA) with a dose of 200 mg × kg^−1^ body weight (*n* = 6). Cystometry was performed 48 h after CYP injection. We evaluated the effect of CaSR agonist on CYP-induced bladder hyperactivity via intravesical infusion with saline followed by intravesical infusion with 1 mM CaSR agonist.

### 4.10. Data and Statistical Analysis

GraphPad Prism 6 (GraphPad Software Inc., San Diego, CA, USA) was used for graphing and statistical analysis. The investigator who executed data analysis was blinded to the experimental groups. All values were expressed as the mean ± standard deviation (SD). Differences before and after different treatments in each group were analyzed using two-tailed paired Student’s *t*-test. Parameters were compared using one-way analysis of variance (ANOVA) followed by Tukey’s post hoc tests to assess differences among groups. Two-way ANOVA followed by Sidak’s post hoc tests was applied to analyze differences among groups with multiple treatments. The post hoc tests were conducted only if F in ANOVA achieved statistical significance (*p* < 0.05) and there was no significant variance in homogeneity. Values of *p* < 0.05 indicated a statistical significance.

## Data Availability

The data presented in this study are available on request from the corresponding author. Authors can confirm that all relevant data are included in the article.
